# Heat shock protein 40 enhances axon regeneration in a mouse model of traumatic optic neuropathy

**DOI:** 10.4103/NRR.NRR-D-25-00034

**Published:** 2026-03-14

**Authors:** Jiaxing Wang, Ying Li, Felix L. Struebing, Sandra Jardines, Su-Ting Lin, Fangyu Lin, Eldon E. Geisert

**Affiliations:** 1 Department of Ophthalmology, Emory University, Atlanta, GA, USA; 2 Center for Neuropathology and Prion Research, Ludwig Maximilian University of Munich, Munich, Germany

**Keywords:** axon regeneration, complex trait, *Dnajc3*, genomics, heat shock protein 40, mouse, optic nerve, retinal ganglion cell

## Abstract

Retinal ganglion cell death occurs following injury to the optic nerve either by trauma or in disease such as glaucoma, leading to severe vision loss. Recent innovations have demonstrated that optic nerve regeneration is feasible; however, the regeneration is limited. The aim of the present study is to identify genomic elements enhancing axon regeneration. We have taken a forward genetics approach using the BXD recombinant mouse strains to identify a gene that increases the extent of optic nerve regeneration. Axon regeneration was induced by knocking down *Pten* in retinal ganglion cells using adeno-associated virus to deliver an shRNA followed by an intravitreal injection of Zymosan with CPT-cAMP that produced a mild inflammatory response. Retinal ganglion cell axons were damaged by optic nerve crush. Following a 12-day survival period, regenerating axons were labeled by intravitreal injection of Cholera Toxin B conjugated with Alexa Fluor 647. Two days later, labeled axons within the optic nerve were examined to determine the number of regenerating axons and the distance they traveled down the optic nerve. The analysis revealed a surprising difference in the amount of axonal regeneration across all 33 BXD strains. There was a 7.5-fold difference in the number of regenerating axons and a 4-fold difference in the distance traveled by regenerating axons. These data were used to generate an interval map defining genomic loci that modulate enhanced axonal regeneration. A quantitative trait locus modulating axon regeneration was identified on Chromosome 14 (115 to 119 Mb). Within this locus were 16 annotated genes. Subsequent testing revealed that one candidate gene, *Dnajc3*, modulated axonal regeneration. *Dnajc3* encodes heat shock protein 40 (HSP40), a molecular chaperone. Knocking down *Dnajc3* in the high regenerative strain (BXD90) led to a decreased regeneration response, whereas, overexpression of *Dnajc3* in a low regenerative strain (BXD34) resulted in an increased regeneration response. These findings reveal that *Dnajc3* not only increases the number of regenerating axons, but also increases the distance that axons travel. The enhanced regeneration will prove to be critical for functional recovery in humans, where the distance axons travel to their targets is considerably longer than that of mice.

## Graphical abstract

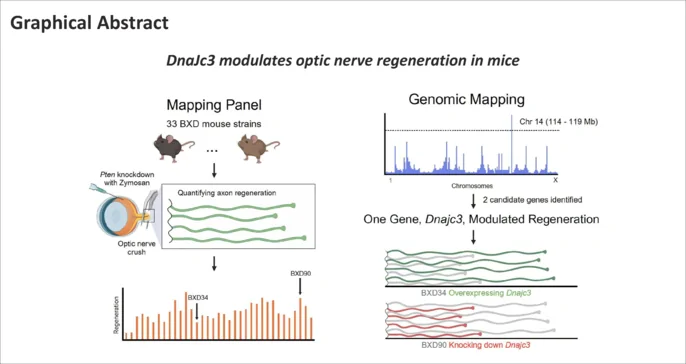

## Introduction

Retinal ganglion cell (RGC) axons are compromised in progressive diseases such as glaucoma (Anderson and Hendrickson, 1974; Quigley et al., 1979; Howell et al., 2007) and also induced by injury in traumatic optic neuropathy (Au and Ma, 2022; Chen et al., 2022; Chalela, 2023; Blanch et al., 2024). This damage to the axons in the optic nerve results in axonal degeneration, RGC death and a loss of visual function. The absence of regeneration is believed to be linked to epigenetics of transcriptional regulation (Moore et al., 2009; Watkins et al., 2013; Apara and Goldberg, 2014; Cheng et al., 2022; Tai et al., 2023; Lukomska et al., 2024). Selected molecular processes can be altered by experimental interventions designed to promote RGC survival and axon regrowth (Park et al., 2008; Moore et al., 2009; Kurimoto et al., 2010; de Lima et al., 2012; Norsworthy et al., 2017). Underpinning the RGC survival and axon regeneration is a cascade of cellular interactions involving multiple molecular pathways (Chen et al., 1997; Wang et al., 2002; Park et al., 2008; Geisert et al., 2009; Moore et al., 2009; Solovieff et al., 2013; Civelek and Lusis, 2014; Belin et al., 2015; Nawabi et al., 2015; Omura et al., 2015; Williams and He, 2016; Barber et al., 2017; Benowitz et al., 2017; Norsworthy et al., 2017; Wang et al., 2021; Schaeffer et al., 2023). Ultimately, these pathways contribute to the survival of injured RGC by modulating processes such as apoptosis (Harder et al., 2012; Fernandes et al., 2013) and autophagy (Park et al., 2008) and altering response to growth factors (Cui et al., 1999; Koprivica et al., 2005; Hoyng et al., 2014; Williams and He, 2016). Regenerating axons in the optic nerve not only require the reactivation of axon growth programs (Benowitz et al., 2017), but also interact with growth inhibitors in the adult central nervous system (CNS). Inhibitory interactions are associated with many glial components within the optic nerve, such as astrocytes (Silver and Miller, 2004; Yiu and He, 2006), oligodendrocytes (Wang et al., 2002), and glial scars (Silver and Miller, 2004; Yiu and He, 2006). Ultimately, the regeneration is promoted by changes in transcriptional regulation (Moore et al., 2009; Watkins et al., 2013; Apara and Goldberg, 2014; Cheng et al., 2022; Tai et al., 2023; Lukomska et al., 2024) and signaling cascades (Park et al., 2008; Lorber et al., 2009; Galvao et al., 2018; Xia et al., 2024). Given the complex nature of axonal regeneration, it is difficult to define the combination of events that would enhance the current protocols inducing axonal regrowth in the optic nerve.

One approach to facilitating axonal regrowth is to identify differences across inbred mouse strains or genetic backgrounds that enhance regeneration (Omura et al., 2015). Omura et al. (2015) tested 9 different inbred strains and found that axons of the CAST/Ei strain grew further in tissue culture on inhibitory substrates than the other 8 strains. In the intact mouse, the regenerative response of CAST/Ei strain was greater than that observed in the C57BL/6 strain. Unfortunately, using individual inbred mouse strains makes identifying the specific genomic loci driving increased regeneration very difficult. To overcome this problem, our group has taken advantage of the BXD genetic reference panel to identify genomic loci modulating induced optic nerve regeneration (Geisert and Williams, 2020).

In the current study, a forward-genetics approach was used to define genomic loci modulating the axonal regeneration produced by knocking down *Pten* (phosphatase and tensin homolog) (Park et al., 2008; Kurimoto et al., 2010) and inducing a mild inflammatory response (Leon et al., 2000) in mice that received a crush to the optic nerve. The amount of axon regeneration was quantified in 33 BXD recombinant inbred mouse strains. Defining the extent of induced axonal regeneration allowed us to map a quantitative trait locus (QTL) modulating the regrowth of axons down the optic nerve. Within this locus a single gene, *Dnajc3* (encoding heat shock protein 40 [HSP40]) was identified as modulating the indued axonal regeneration. Identifying HSP40 provides a new insight into the regulation of neuronal repair.

## Methods

### Mice

For mapping the locus modulating axonal regeneration, 33 BXD recombinant inbred strains and their parental strains – C57BL/6J (RRID: IMSR_JAX:000664) and DBA/2J (RRID: IMSR_JAX:000671) were used in this study. For each strain, a minimum of four mice were used. All mice were 60–70 days of age at the time of initial treatment (**Additional Table 1**). Controls were run with the C57BL/6J (*n* = 6) and DBA/2J (*n* = 6) mouse strains. For the mapping study, 183 mice were used. The study began in July 2017 and was halted in March 2020 due to COVID-19 restrictions placed on research at Emory University. The study restarted in May 2021 and was completed in September 2022. To minimize variability in surgical technique and analytical analysis, all of the surgeries and quantification were conducted by one individual (JW). As data collection was interrupted, we examined the data to determine whether there was a systematic batch effect in the results. The first group of animals was the cohort that appeared in the publication by Wang et al. (2018). This was compared to the remaining animals that were divided into two groups. Thus, we have an early group, an intermediate group, and the last group of mice. When we compared the regenerative response of each group to the other two groups, there was no statistically significant difference between all four measures used (number of axons at 0.5 mm and 1 mm from the crush site, distance of the 5 lonest axons, and longest single axon). The early group was not different from the intermediate group or the last group, and the intermediate group was not different form the last group. We also examined the animals before and after COVID-19 and again there was no significant difference in the regenerative response. For testing candidate genes, we chose the BXD90 (*n* = 21) strain, which has a high regenerative response, and the low regenerating mouse strain was BXD34 (*n* = 17). We used these two strains to check the regeneration after modulating candidate genes. We also examined one parental strain C57BL/6J (*n* = 20).

**Additional Table 1:** Measurements used to quantify axonal regeneration in BXD mouse strains (Strains)

Strain	N	Count 0.5	Count 1.0	Distance 5 axons (mm)	Distance 1 axon (mm)
C57BL/6J Control	6	0 ± 0	0 ± 0	0.2 ± 0.1	0.4 ± 0.1
DBA/2J Control	6	8 ± 21	0 ± 0	0.2 ± 0.1	0.4 ± 0.1
C57BL/6J	5	356 ± 76	2 ± 3	0.9 ± 0.2	1.2 ± 0.3
DBA/2J	8	613 ± 138	5 ± 2	1.1 ± 0.2	1.3 ± 0.1
BXD11	4	277 ± 32	1 ± 1	0.8 ± 0.1	1.2 ± 0.2
BXD13	4	180 ± 132	1 ± 1	0.5 ± 0.2	0.9 ± 0.2
BXD16	5	348 ± 256	3 ± 5	0.8 ± 0.5	1.3 ± 0.7
BXD18	5	209 ± 65	0 ± 1	0.6 ± 0.1	0.9 ± 0.2
BXD22	4	249 ± 131	3 ± 3	0.7 ± 0.4	1.4 ± 0.6
BXD27	4	794 ± 250	10 ± 5	1.7 ± 0.5	2.0 ± 0.4
BXD28	5	261 ± 40	6 ± 2	1.2 ± 0.2	1.4 ± 0.4
BXD29Tlr4	4	760 ± 158	13 ± 1	2.0 ± 0.4	2.4 ± 0.3
BXD31	4	511 ± 134	6 ± 1	1.3 ± 0.2	1.5 ± 0.3
BXD34	4	135 ± 121	1 ± 2	0.5 ± 0.2	0.9 ± 0.2
BXD38	4	425 ± 100	3 ± 2	1.0 ± 0.2	1.2 ± 0.1
BXD40	8	322 ± 111	3 ± 2	1.0 ± 0.2	1.2 ± 0.3
BXD43	6	225 ± 51	1 ± 1	0.8 ± 0.2	1.1 ± 0.2
BXD48	7	325 ± 125	3 ± 4	0.9 ± 0.5	1.3 ± 0.4
BXD56	6	443 ± 180	3 ± 3	0.9 ± 0.2	1.3 ± 0.4
BXD51	6	245 ± 62	1 ± 1	0.8 ± 0.1	1.0 ± 0.1
BXD50	7	503 ± 145	5 ± 5	1.0 ± 0.3	1.5 ± 0.7
BXD63	4	557 ± 86	8 ± 2	1.1 ± 0.1	1.4 ± 0.2
BXD65	4	348 ± 166	5 ± 4	1.1 ± 0.4	1.5 ± 0.5
BXD67	5	877 ± 108	14 ± 6	1.5 ± 0.2	1.9 ± 0.3
BXD69	6	641 ± 135	9 ± 4	1.4 ± 0.3	1.8 ± 0.2
BXD71	5	674 ± 136	10 ± 3	1.8 ± 0.4	2.1 ± 0.3
BXD73	4	563 ± 139	12 ± 3	1.3 ± 0.2	1.9 ± 0.5
BXD74	5	652 ± 242	6 ± 4	1.2 ± 0.5	2.0 ± 0.6
BXD75	4	243 ± 96	4 ± 2	1.1 ± 0.2	1.3 ± 0.2
BXD85	5	357 ± 60	3 ± 3	0.9 ± 0.2	1.3 ± 0.2
BXD86	6	240 ± 176	1 ± 2	0.6 ± 0.3	1.1 ± 0.5
BXD87	5	456 ± 45	3 ± 3	0.9 ± 0.2	1.4 ± 0.5
BXD90	7	1030 ± 160	19 ± 5	1.5 ± 0.4	2.1 ± 0.7
BXD99	6	806 ± 179	13 ± 4	1.3 ± 0.3	1.7 ± 0.5
BXD102	5	236 ± 55	1 ± 0	0.8 ± 0.1	1.1 ± 0.1

The measurements include the number of axons at 0.5 mm from the crush (Count 0.5) and 1mm from the crush site (Count 1.0), along with the distance traveled for the 5 longest axons (Distance 5 axons) and the single longest axon (Distance 1 Axon). The number of cases for each strain is shown (N). Measurements are shown as mean ± SD.

All mice were obtained from Jackson Laboratory (Bar Harbor, ME, USA). The mice were housed in a pathogen-free (level 1) facility at Emory University, maintained on a 12-hour light/dark cycle, provided with food and water *ad libitum*, and temperature was maintained between 18–24°C with relative humidity between 30% and 70%. Mice were housed in group cages ranging from two to five mice per cage. All procedures involving animals were approved by the Animal Care and Use Committee of Emory University (Protocol Number, PROTO201900198, July 8, 2024) and were in accordance with the ARVO Statement for the Use of Animals in Ophthalmic and Vision Research.

### Surgery

The optic nerve regeneration protocol developed by others (Park et al., 2008; Kurimoto et al., 2010; de Lima et al., 2012) was used to induce regeneration after optic nerve crush (ONC). The detailed protocol was described in our previous publication (Wang et al., 2018). Briefly, the treatment included knocking down of *Pten a*nd intravitreal injection of Zymosan plus CPT-cAMP. We used AAV-sh*Pten*-GFP (*Pten* short hairpin RNA-GFP packaged into adeno-associated virus 2 backbone constructs, titer = 1.5 × 10^12^ vg/mL) to knock down *Pten*. The shRNA target sequence is 5′-AGG TGA AGA TAT ATT CCT CCA A-3′ as described by Zukor et al. (2013). The efficient suppression of Pten expression in the RGCs by this *Pten* shRNA has been proven in a previous study (Wang et al., 2018). For all surgeries, the mice were deeply anesthetized via intraperitoneal injection with a mixture of 15 mg/kg of xylazine and 100 mg/kg of ketamine. Two weeks prior to ONC, the mice were deeply anesthetized and injected intravitreally using a transpupillary approach (Lin et al., 2025) with 2 µL of AAV-sh*Pten*-GFP. The efficiency of the *Pten* knockdown was demonstrated by examining the phosphor-S6 staining in the cells transduced by the AAV-sh*Pten*-GFP vector (Park et al., 2008; Lim et al., 2016). When retinas were transduced with AAV-sh*Pten*-GFP, all RGCs labeled with GFP also expressed high levels of Phospho-S6 (**Additional Figure 1**). Optic nerve crush was performed as described by Templeton and Geisert (Templeton and Geisert, 2012). Briefly, under the binocular operating scope, a small incision was made in the conjunctiva. The optic nerve was visualized and then crushed 1 mm behind the eye with Dumont N7 angled crossover tweezers for 5 seconds, avoiding injury to the ophthalmic artery. Immediately following ONC, Zymosan (Sigma, St. Louis, MO, USA, Z4250, Lot# BCBQ8437V), along with the cAMP analog CPT-cAMP (Sigma, C3912, Lot# SLBH5204V, total volume 2 µL) was injected into the vitreous to induce an inflammatory response and augment regeneration. The animals were allowed to recover from anesthesia and returned to their cages. Twelve days after ONC (2 days before sacrifice), the animals were deeply anesthetized, and Alexa Fluor® 647-conjugated Cholera Toxin B (CTB; ThermoFisher, Waltham, MA, USA, Cat# C34778) was injected into the vitreous for labeling of the regenerated axons. All the intravitreal injections and optic nerve crushes were performed by one well-trained postdoctoral fellow to avoid technical variations during the surgical procedure. At 14 days after ONC, the mice were deeply anesthetized and perfused through the heart with phosphate-buffered saline (PBS; pH 7.3) followed by 4% paraformaldehyde in phosphate buffer (pH 7.3).

### Preparation of the optic nerve

The optic nerves of the crushed side (right eye), along with optic chiasms and brains, were dissected and post-fixed with 4% paraformaldehyde in phosphate buffer overnight. The optic nerve was cleared with FocusClear^TM^ (CelExplorer, Hsinchu, Taiwan, China) for up to 4 hours until totally transparent. A small chamber was built on the slide to provide enough space for the whole nerve thickness and to keep the nerve from being damaged from flattening. The optic nerve was then mounted in the chamber using MountClear^TM^ (CelExplorer) and the slides were cover-slipped. FocusClear^TM^ has been used to clear brain tissue for whole brain imaging (Moy et al., 2015) as well as clearing of the optic nerve of transgenic zebrafish to observe axon regeneration (Diekmann et al., 2015). It allowed us to scan the whole thickness of the optic nerve for better understanding of the status of axon regeneration, while providing clear imaging of regenerated axons from the optical slices scanned via confocal microscopy for counting. It also allowed us to determine the longest five axons or longest single axon along the nerve from z-stack of the whole nerve.

### Quantitation of axon regeneration

Cleared optic nerves were examined using a Nikon Eclipse Ti2 confocal microscope (Melville, NY, USA) by scanning through individual optical slices. Green pseudo-color was used for CTB-labeled axons in all the optic nerve images of this study for clear visual observation. Stacked images were taken at 10 µm increments, a total of 20–50 optical slices for each optic nerve.

For quantifying the number of axons, we calculated the virtual thickness of an optical slice from the confocal microscope. As previously described by Wang et al. (2018), we determined that the thickness of the optical section was 6 µm where refractive index (n) was 1.517, the numerical aperture (Na) was 0.45 and the excitation wavelength was 637 nm. Since the optical section was 6 µm and the spacing between optical sections was 10 µm, single axons were not counted multiple times.

**Figure 1:**
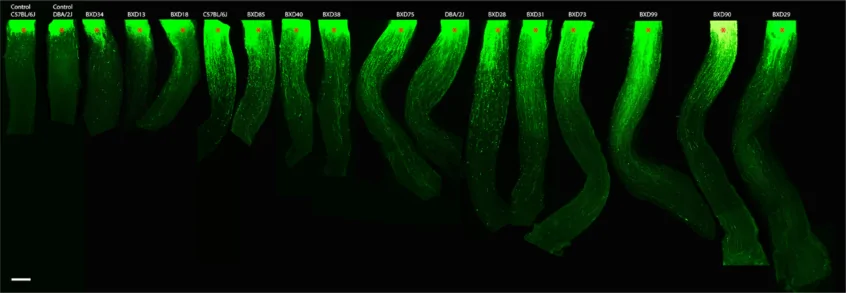
A series of photomicrographs from 17 optic nerves selected from 15 different strains of mice. The two images to the left are from control mice with optic nerve crush only (Control C57BL/6J and Control DBA/2J). The remaining nerves are from animals in which the optic nerve was crushed and regeneration treatments were applied. Red asterisks represent the crush site. The strains from left to right are: C57BL/6J crush only, DBA/2J crush only, BXD34, BXD13, BXD18, C57BL/6J, BXD85, BXD40, BXD38, BXD75, DBA/2J, BXD28, BXD31, BXD73, BXD99, BXD90 and BXD29. Scale bar: 200 µm.

The number of CTB-labeled axons at 0.5 mm from the crush site were counted in at least six sections per case and calculated by the equation (Σad=πr^2^ × [average axons/mm]/t) as described by Leon et al. (2000). The cross-sectional width of the nerve was measured at the point at which the counts were taken and was used to calculate the number of axons per millimeter of nerve width. The total number of axons extending distance d in a nerve having a radius of *r*, was estimated by the summation of all sections. Since we used confocal images instead of longitudinal cross-sections described in previous studies (Kurimoto et al., 2010; de Lima et al., 2012), the optical resolution in z (0.5 µm) was considered as the t (thickness of the slide) in the equation. The number of axons at 0.5 mm from the crush site, the number of axons at 1 mm from the crush site, the distance traveled by the five longest axons and the length of the longest single axon were all measured (see **Figure 1** of our previous publication (Wang et al., 2018)).

### Interval mapping

The axon regeneration data were subjected to conventional QTL analysis using simple and composite interval mapping using the mm10 assembly. Genotypes were regressed against each trait using the Haley-Knott equations implemented in the WebQTL module of GeneNetwork (Wang et al., 2003; Overall et al., 2009). Empirical significance thresholds of linkage were determined by permutations (Churchill and Doerge, 1994). We generated interval maps for all four regeneration measures: number of axons at 0.5 mm from the crush site, number of axons at 1 mm from the crush site, distance traveled by the five longest axons and the length of the longest single axon. In the present study, we made a synthetic trait for axon regeneration by combining all four datasets into a single measure and used this to produce an interval map. To identify loci, and also to nominate candidate genes, we used the following approaches: interval mapping for the traditional phenotypes, candidate gene selection within the QTL region, cis-eQTL analysis of gene expression, and trans-eQTL analysis.

### Adeno-associated virus vector

To knock down the genes of interest, we used a similar AAV vector to the *Pten* knockdown as previously described (Wang et al., 2018). Briefly, short hairpin RNA was designed for genes of interest, including *Dnaj* heat shock protein family member 40 (*Dnajc3*; NM_008929.3) and UDP-glucose glycoprotein glucosyltransferase 2 (*Uggt2*; NM_001081252.2), using the shRNA-designer from Biosettia (https://biosettia.com/support/shrna-designer/). The targeting sequence was selected on the exon region that contained no single nucleotide polymorphisms (SNPs) between C57BL/6J and DBA/2J strains. The shRNA sequence for the gene of interest was inserted into the AAV-sh*Pten*-GFP replacing the shRNA sequence for *Pten*. The transfection efficiency of these vectors was the same as that of AAV-sh*Pten*-GFP, which was tested previously (Wang et al., 2018). For AAV-sh*Dnajc3*-GFP, the shRNA target sequence is 5′-GCA ACC AGC AAA TAT GAA T-3′, virus titer = 1.6 × 10^14^ vg/mL. For AAV-sh*Uggt2*-GFP, the shRNA target sequence is 5′-GCC TGG GAT TAT CAG CAA T-3′, virus titer = 1.4 × 10^14^ vg/mL. For the overexpression of *Dnajc3* in the RGCs, the mRNA sequence of *Dnajc3* (NM_008929.4) was packed into the AAV vector. Controls were injected with AAV-GFP vector. The virus titer was 4 × 10^12^ genomes. All of our plasmids were made at Emory Integrated Genomic Core (https://cores.emory.edu/eigc/) and were packed into AAV2 at Emory Viral Vector Core (https://neurology.emory.edu/ENNCF/viral_vector/). Previously, we have demonstrated that the AAV transduces approximately 54% of the RGCs in mice (Wang et al., 2018).

### Testing candidate genes by knockdown or overexpression

Candidate genes were tested in our model optic nerve regeneration system. The AAV vector, either with shRNA for knocking down a gene or with full-length sequence for overexpressing a gene, was injected into the vitreous chamber 1 week before the start of the regeneration protocol. The vectors included: AAV-sh*Uggt2*-GFP and AAV-sh*Dnajc3*-GFP for knockdown, or AAV-*Dnajc3* for overexpression. For testing the effects of knocking down candidate genes, we used a BXD strain that demonstrated robust regeneration, BXD90. The BXD90 mice received an intravitreal injection of either AAV-sh*Uggt2*-GFP or AAV-sh*Dnajc3*-GFP. For testing the effects of overexpressing a gene, we used either a low regenerating strain, BXD34, or the parental strain, C57BL/6J. The AAV-*Dnajc3* vector was injected into the intravitreal chamber one week before the initiation of the regeneration protocol. The same strain of mice receiving AAV-GFP was used as a control group for both the knockdown and overexpression experiments.

### Immunohistochemistry

Mice were deeply anesthetized with ketamine (100 mg/kg) and xylazine (10 mg/kg), and perfused with PBS followed by 4% paraformaldehyde in phosphate buffer (pH 7.3). Eyes were removed, and the retinas were carefully dissected and prepared as flat-mounts following a previously described protocol (King et al., 2018). The retinas were blocked in PBS containing 3% bovine serum albumin (BSA) (Sigma), 3% donkey serum, and 0.5% Triton X-100 (Sigma). The primary antibodies were diluted in blocking buffer and include: guinea pig anti-RBPMS (PhosphoSolutions, Aurora, CO, USA, Cat# 1830), UGGT2 (Fisher Scientific, Waltham, MA, USA, Cat# PIPA527960), DNAJC3 (Proteintech, Rosemont, IL, USA, Cat# 26721-AP), GFP (MilliporeSigma, Burlington, MA, USA, Cat# AB16901), S6 (Cell Signaling Technology, Danvers, MA, USA, Cat# 2217), and Phospho-S6 (Cell Signaling Technology, Cat# 2211). All primary antibodies were diluted to 1:500 with the exception of phospho-S6 (1:1000). The retinas were incubated with primary antibodies for 48 hours at 4°C. After incubation, retinas were rinsed three times in PBST (PBS containing 0.1% Tween-20), and then incubated overnight at 4°C with the appropriate secondary antibodies (1:1000; Jackson ImmunoResearch, West Grove, PA, USA). Secondary antibodies used included: Alexa Fluor 488 AffiniPure donkey anti-rabbit (Cat# 715-545-152), Alexa Fluor 594 AffiniPure donkey anti-rabbit (Cat# 711-585-152), Alexa Fluor 594 AffiniPure donkey anti-guinea pig (Cat# 706-585-148), Alexa Fluor 647 AffiniPure donkey anti-guinea pig (Cat# 706-605-148), Alexa Fluor 488 AffiniPure donkey anti-chicken (Cat# 703-545-155). For nuclear counterstaining, TO-PRO-3 (thiazole red, Thermo Fisher Scientific, Waltham, MA, USA, Cat# T3605) was used to label all nuclei within the ganglion cell layer. Imaging was performed using a Nikon Eclipse Ti2 microscope equipped with an A1R confocal scanning system (Nikon, Inc., Melville, NY, USA).

### Statistical methods used in this study

Data are presented as Mean ± SE (Standard Error of the Mean). All differences in axon counts and regeneration distances between the strains were analyzed by Exact Wilcoxon-Mann-Whitney Test Calculator (Marx et al., 2016) (https://ccb-compute2.cs.uni-saarland.de/wtest/?id=www/www-ccb/html/wtest,). A value of *P* < 0.05 was considered statistically significant.

## Results

**Figure 2:**
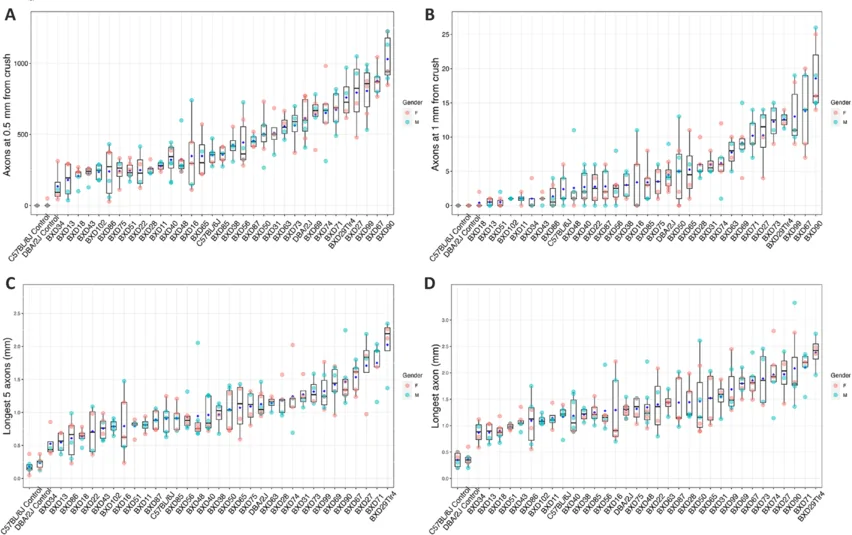
Axon regeneration measurement across all strains. The number of axons at 0.5 mm from the crush site (A), the number of axons 1 mm from the crush site (B) are shown. In addition, the distance traveled by the longest five regenerating axons (C) and the longest single axon (D) are illustrated, in two control strains (C57BL/6J and DBA/2J untreated mice) and 33 strains that went through the regeneration protocol. Each animal is represented by a single dot, with the pink dots for females and the light blue dots for males. Comparing the strains with the greatest regeneration with the strains with the least regeneration, there is a 7.5-fold difference in the number of axons at 0.5 mm from crush site (A) and 7.5-fold difference at 1 mm from crush site (B). There is an approximate 4-fold difference in the distance of the longest five axons and 3-fold difference in the distance of the longest single axon, when comparing the strains with the longest axons to the ones with the shortest axons. The fact that the parental strains are not the extremes of the distributions indicates that there is genetic transgression in axonal regeneration, suggesting a minimum two genomic loci modulating the phenotype of axon regeneration.

Axon regeneration was examined in 33 strains of mice (31 BXD strains and two parental strains, C57BL/6J and DBA/2J). Of these 33 strains, the data from nine were used in a previous study demonstrating that optic nerve regeneration is a complex trait (Wang et al., 2018). The BXD strain set has proven to be a valuable genetic reference panel for vision research (Geisert and Williams, 2020). Here, we examined the extent of axon regeneration 14 days following optic nerve crush. There was a surprising difference in regenerative capacity that was dependent upon the specific BXD strain examined (**Figure 1**). Some strains (BXD13, BXD18, and BXD34) showed very little axon regeneration; while other strains (BXD99, BXD90, and BXD29) displayed a significant amount of axon regrowth down the optic nerve (**Figure 1**). In all cases, optic nerve crush followed by the axon regeneration treatment led to more axonal regrowth than observed in the two control cases (C57BL6J and DBA/2J), which received optic nerve crush only (**Figure 1**). The axon regeneration for each strain was quantified by counting the number of regenerating axons at 0.5 mm (**Figure 2A**) and 1 mm (**Figure 2B**) from the crush site (**Additional Table 1**). The distance the axons had traveled down the optic nerve was also quantified by measuring the length of the longest five axons (**Figure 2C**) and the distance traveled by the longest single axon (**Figure 2D**). These data were compared to axon regeneration in control mice that did not receive the regeneration treatment (**Figure 2**). When examining the regenerative capacity of the different BXD strains, the number of regenerating axons at 0.5 mm varied by 7.5-fold, with the lowest number in strain BXD34 being an average of 135 axons and the greatest number of regenerating axons in strain BXD90 being an average of 1030 axons (**Figure 2A**). A similar pattern is observed 1 mm from the crush site, with the lowest average number of regenerating axons in BXD18 and the highest average number of regenerating axons in BXD90 (**Figure 2B**). When examining the distance axons have regenerated down the optic nerve, the general pattern is similar to that observed for the number of regenerating axons. The fold change in axon growth for the longest five regenerating axons was 4 with the shortest axons in strain BXD34 and the longest axons in BXD29 (**Figure 2C**). This is also the case for the longest single axon with the shortest strain being BXD34 and the longest being BXD29 (**Figure 2D**). The data suggest that the number of regenerating axons and the distance the axons travel down the optic nerve reflect a similar genetic underpinning for the strains with the fewest axons also have the shorter regenerating axons. It is also the case that the strains with the greatest number of axons also have axons that travel the greatest distance down the optic nerve.

### Interval mapping

**Figure 3:**
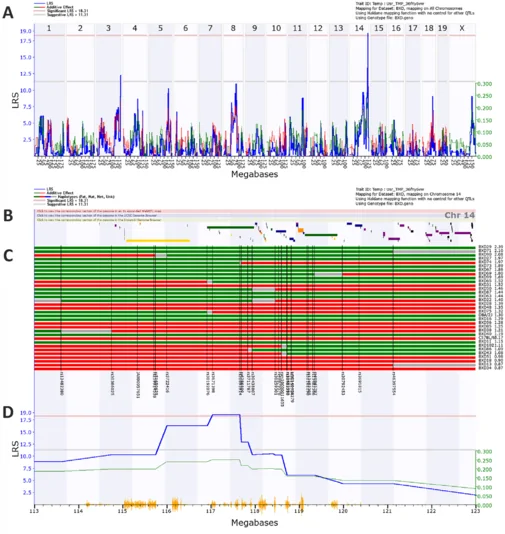
Genome-wide quantitative loci analysis of optic nerve regeneration. A genome-wide interval map of axon regeneration (A). The interval map plots the likelihood ratio statistic (LRS) across the genome from chromosome 1 to chromosome X. The light gray line is the suggestive level and the light red line is the genome-wide significance (*P* = 0.05). When the optic nerve regeneration measures were mapped to the mouse genome, there was a significant association between optic nerve regeneration and a locus on Chromosome 14. A map of gene locations across Chr 14 from 113 to 123 Mb is shown (D). The locations of genes within this region are schematically shown in the top panel (B). The haplotype map for the 33 strains in the optic nerve regeneration dataset is illustrated in the middle panel with each strain indicated on the right (C). Red represents the B6 alleles, green defines the D2 alleles, blue represents regions of the DNA that are heterozygotic and gray is unmapped. The genomic markers used in the mapping process are listed at the bottom of the haplotype map. At the far right is a list of the specific BXD strains and the associated regeneration measurements going from the most regeneration at the top to the least at the bottom. A quantitative trait locus (QTL) map of regeneration on Chr 14 is shown below the haplotype map. An LRS above the pink line is statistically significant (*P* < 0.05). A positive additive coefficient (green line) indicates that D2 alleles are associated with higher trait values. The significant peak for optic nerve regeneration is from 114 to 119 Mb. Vertical orange lines at the bottom of the plot show the single nucleotide polymorphisms on Chr 14.

These data were used to map genomic loci that may modulate the regenerative response in the BXD strains. The unbiased, forward genetics approach was used to create a genome-wide interval map for each of the measures of regeneration: number of axons 0.5 mm from the crush site, number of axons 1 mm from the crush site, distance traveled by the five longest axons and the length of the longest single axon (**Additional Figure 2**). All four interval maps showed a similar pattern with a large peak being on distal chromosome (Chr) 14. The peak on Chr 14 (115 Mb to 119 Mb) is above the suggestive level for all measures. The map on the longest single axon has a QTL that reached the significance level (**Additional Figure 2D**). Since all of the genome wide scans were similar, all of these data were combined to create a synthetic trait, “axonal regeneration”. The genome-wide scan for axonal regeneration revealed the same significant peak on Chr 14 (**Figure 3**). For further analysis, we used data from the combined synthetic trait, axon regeneration, to identify the QTL modulating axon regeneration as well as defining the candidate genes within the region.

### Candidate genes

**Figure 4:**
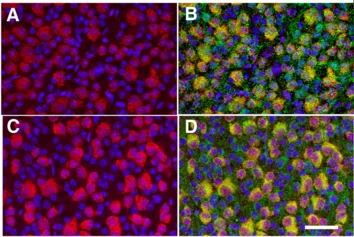
Expression of HSP40 and UGGT2 in RGCs. Retinal flat mounts were stained with RBPMS (red) and TO-PRO-3 (blue) to label nuclei (A and C). The retinas were also stained with a green HSP40 (B) or UGGT2 (D). The staining for HSP40 or UGGT2 is illustrated in a merged image (B and D). Both HSP40 and UGGT2 display a strong cytoplasmic labelling pattern commensurate with their roles in the endoplasmic reticulum. Scale bar: 50 µm. HSP40: Heat shock protein 40; RGC: retinal ganglion cell; UGGT2: UDP-glucose glycoprotein glucosyltransferase 2.

The interval map for the longest single axon and the synthetic trait axon regeneration both had a significant QTL peak on Chr 14. To identify genes modulating axonal regeneration in the BXD strains, we examined a 4 Mb region around the peak, ranging from 115 Mb to 119 Mb (**Figure 3**). Within this region, there were 16 annotated genes: *Mir18*, *Mir19a*, *Mir20a*, *Mir17hg*, *Gpc5*, *Gpc6*, *Gm19845*, *Dct*, *Tgds*, *Gpr180*, *Sox21*, *Abcc4*, *Cldn10*, *Dzip1*, *Dnajc3* and *Uggt2*. In general, ideal candidate genes should have a nonsynonymous SNP between the two parental strains that affects protein sequence and function, or there should be genomic elements with cis-QTLs affecting expression levels. Of the 16 genes within the region, two had nonsynonymous SNP: *Abcc4* and *Cldn10*. To determine if any of these amino acid changes would affect protein function, we ran a SIFT analysis (Choi et al., 2012). All of the changes in both genes were tolerated and should not disrupt protein function. Thus, these two genes did not appear to be good candidates and were removed as potential candidate genes. Two genes were found with cis-QTLs: *Dnajc3* and *Uggt2*. The difference in expression levels of these two candidate genes was confirmed by examining RNAseq comparisons between the parental strains (Wang et al., 2019). Both of these genes are expressed in the cytosol of the RGC (**Figure 4**). This is consistent with their role in the endoplasmic reticulum. Furthermore, if we examine the single cell RNAseq datasets of mouse RGCs (Tran et al., 2019), all RGC subtypes express both *Dnajc3* and *Uggt2*. These data indicate that both *Dnajc3* and *Uggt2* are good candidate genes for modulating the response of RGCs to axonal injury.

### Knockdown of *Dnajc3* and *Uggt2*

**Figure 5:**
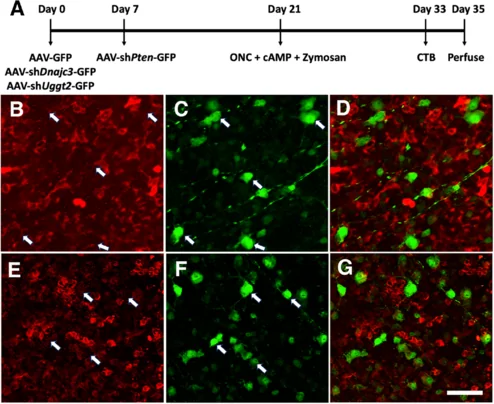
Down-regulation of *Dnajc3* and *Uggt2*. The modified regeneration protocol is illustrated in A. 7 days prior to the knockdown of *Pten* (day 0), AAV-GFP (control), AAV-*Dnajc3*-GFP or AAV-sh*Uggt2*-GFP was injected into the eye. On day 7, AAV-sh*Pten*-GFP was then injected into the eye. Two weeks later (day 21), the optic nerve is crushed and Zymosan and cAMP were injected into the eye. On day 33, the eye was injected with Alexa Fluor® 647-conjugated Cholera Toxin B and 2 days later, the animal was perfused. In B–D, flatmount of a retina injected with AAV-sh*Dnajc3*-GFP are shown. In the first panel B, the staining for HSP40 (*Dnajc3*) is shown labeling RGCs. (C) The labeling for GFP. Notice the lack of HSP40 staining in the GFP expressing RGCs (white arrows in B and C). (D) A merged image of B and C. A similar result was observed when AAV-sh*Uggt2* was used (E–G). Immunostaining for UGGT2 (E) revealed that the RGCs transduced with sh*Uggt2* and labeled with GFP do not express UGGT2 (arrows in E and F). The merged image is shown in G. Scale bar: 50 µm. AAV: Adeno-associated virus; Dnajc3: *Dnaj* heat shock protein family member 40; GFP: green fluorescent protein; HSP40: heat shock protein 40; RGCs: retinal ganglion cells; Uggt2: UDP-glucose glycoprotein glucosyltransferase 2.

To further investigate the roles of the two candidate genes in optic nerve regeneration, we extended our study using the existing regeneration model. As an initial test, we chose one of the high regenerative strains, BXD90, to examine the effects of knocking down gene expression using AAV delivery of shRNA. AAV-shRNA-GFP directed against either *Uggt2* or *Dnajc3* was injected into the vitreous chamber of the mouse eye, knocking down the expression of each candidate gene. The injection was given 1 week prior to *Pten* knockdown (**Figure 5A**). This allowed the effective transduction of cells with AAV carrying candidate gene-specific shRNA. This also produced a decrease in gene expression of either *Uggt2* or *Dnajc3* prior to the initiation of the regeneration protocol, allowing for the same sequence of our established regeneration experimental protocol. The control group received adeno-associated virus expressing green fluorescent protein (AAV-GFP) at the same time point. Immunohistochemistry was used to demonstrate the successful knockdown of the *Uggt2* and *Dnajc3* in RGCs of the mouse retina (**Figure 5**).

**Figure 6:**
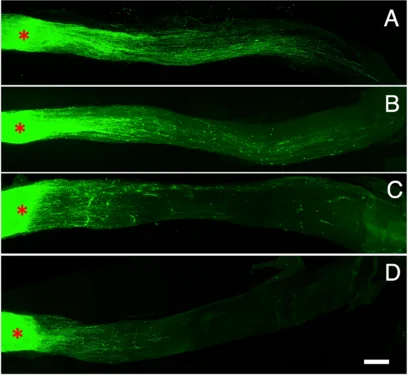
Axon regeneration after Dnajc3 and Uggt2 knockdown. To test for the ability of *Uggt2* and *Dnajc3* to modulate axonal regeneration, we knocked down each of these genes in RGCs of a high regenerating strain BXD90. The location of the crush site is designated by a red asterisk. An optic nerve of a BXD90 strain transduced with AAV-GFP is shown in A. Notice the robust regenerative response. When the retina was transduced with AAV-shRNA-*Uggt2*-GFP there was no noticable decrease in the amount of axon regrowth (B). Knocking down *Dnajc3* with AAV-shRNA-*Dnajc3*-GFP did decrease the number of regenerating axons and the distance they traveled down the nerve (C). For comparison, the nerve from a low regenerating strain, BXD18 is shown in D. The scale bar in D represents 200 µm. AAV: Adeno-associated virus; Dnajc3: *Dnaj* heat shock protein family member 40; GFP: green fluorescent protein; RGCs: retinal ganglion cells; Uggt2: UDP-glucose glycoprotein glucosyltransferase 2.

**Figure 7:**
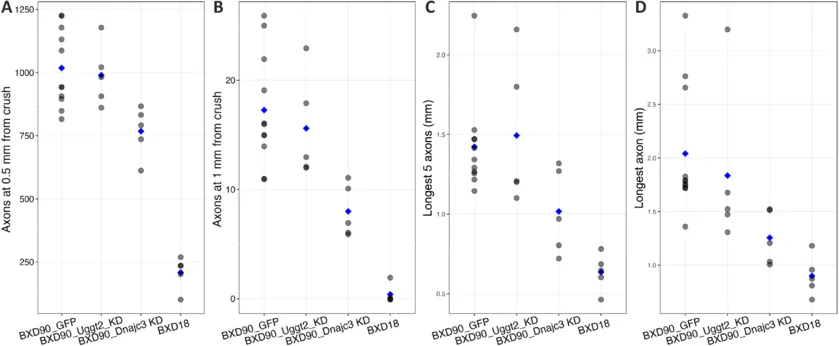
Measurement of axon regeneration after *Dnajc3* and *Uggt2* knockdown in BXD90. BXD90 was one of the strains with robust optic nerve regeneration induced by our regeneration protocol. We used AAV-shRNAs to knockdown the two candidate genes, *Uggt2* and *Dnajc3*, in RGCs prior to testing for optic nerve regeneration. Each animal is represented by a dot and the means are presented as blue diamonds. In A, the number of axons at 0.5 mm from the crush site is shown. The first column is the data from control mice receiving AAV-GFP. The effects of knocking down *Uggt2* is shown in the next column (BXD90_*Uggt2*_KD). The data from knocking down *Dnajc3* is shown next (BXD90_*Dnajc3* KD). Notice a significant decrease in the number of axons regenerating (*P* < 0.05 Mann-Whitney *U* test). The data from one of the low regenerating strains (BXD18) is also shown for comparison. Similar results are shown for the number of axons at 1 mm from the crush site (B). This is also the case for the distance axons regrew down the optic nerve, for both the five longest axons (C) and the longest single axon (D). These data demonstrate that knocking down *Uggt2* does not affect axon growth in BXD90. It demonstrates that knocking down *Dnajc3* can modulate the amount of axon regeneration. Thus, Dnajc3 is the best candidate at the chromosome (Chr) 14 locus for modulating axonal regeneration. Dnajc3: *Dnaj* heat shock protein family member 40; GFP: green fluorescent protein; Uggt2: UDP-glucose glycoprotein glucosyltransferase 2.

When *Uggt2* was knocked down in a high regenerating strain (BXD 90), it did not appear that there was a decrease in axonal regeneration (**Figure 6A** and **B**). Knocking down *Dnajc3* in the BXD90 strain did appear to decrease the amount of axonal regeneration (**Figure 6A** and **C**). This decrease in axonal regrowth did not reach the level of that observed in the BXD18 strain that demonstrated low levels of induced regeneration (**Figure 6D**). These differences were quantified in multiple mice and the data is presented in **Figure 7**. Selectively knocking down *Uggt2* in RGCs did not significantly impact optic nerve regeneration (**Figure 7A–D**), making it unlikely that *Uggt2* modulates axon regeneration in the BXD strains. Knocking down of *Dnajc3* in RGCs decreased the number of axons regenerating in the optic nerve (**Figure 7A** and **B**) as well as the distance that the axons traveled down the optic nerve (**Figure 7C** and **D**) after regeneration treatment for ONC. Quantifying the regenerative capacity revealed a significant 24.6% decrease (*P* < 0.05) in the number of axons at 0.5 mm from the injury site compared with the AAV-GFP control group. There was also a significant 53.7% decrease (*P* < 0.05) at 1 mm from the crush site. A similar finding was observed in the distance axons regenerated down the optic nerve. There was a 28.5% decrease in the distance of the 5 longest axons in the *Dnajc3* knockdown mice relative to the GFP controls (*P* < 0.05), and for the longest single axon there was a 38.5% decrease in distance traveled (*P* < 0.05). Detailed data is shown in **Additional Table 2**. These data indicate that *Dnajc3* is a good candidate gene for modulating axon regeneration down the optic nerve. Furthermore, this decrease in axonal regeneration is selectively due to knocking down *Dnaj3*, for it did not occur following either an AAV-GFP injection or following AAV-siRNA-Uggt2-GFP injection.

**Additional Table 2:** Measurements used to quantify axonal regeneration following knockdown of *Dnajc3* and *Uggt2,* and overexpression of *Dnajc3*

	Knockdown	n	Mean ± SD	Range
	BXD90_GFP	11	1018 ± 154	816 - 1225
Counts at 0.5mm	BXD90_Uggt2 KD	5	990 ± 123	861 - 1178
	BXD90_Dnajc3 KD	5	768 ± 100	612 - 867
	BXD18	5	209 ± 65	101 - 269
	BXD90_GFP	11	17 ± 5	11 - 26
Counts at 1mm	BXD90_Uggt2 KD	5	16 ± 5	12 - 23
	BXD90_Dnajc3 KD	5	8 ± 2	6 - 11
	BXD18	5	0 ± 1	0 - 2
	BXD90_GFP	11	1.4 ± 0.3	1.1 - 2.2
Longest 5 axons (mm)	BXD90_Uggt2 KD	5	1.5 ± 0.5	1.1 - 2.2
	BXD90_Dnajc3 KD	5	1.0 ± 0.3	0.7 - 1.3
	BXD18	5	0.6 ± 0.1	0.5 - 0.8
	BXD90_GFP	11	2.0 ± 0.6	1.4 - 3.3
Longest 1 Axon (mm)	BXD90_Uggt2 KD	5	1.8 ± 0.8	1.3 - 3.2
	BXD90_Dnajc3 KD	5	1.3 ± 0.3	1.0 - 1.5
	BXD18	5	0.9 ± 0.2	0.7 - 1.2
	**Overexpression**	**n**	**Mean ± SD**	**Range**
	B6_GFP	10	311 ± 231	0 - 632
Counts at 0.5mm	B6 Dnajc3 OE	10	634 ± 285	207 - 1047
	BXD34_GFP	8	152 ± 126	37 - 350
	BXD34_Dnajc3 OE	9	462 ± 250	106 - 842
	B6_GFP	10	2 ± 2	0 - 5
	B6 Dnajc3 OE	10	6 ± 3	0 - 10
Counts at 1mm	BXD34_GFP	8	1 ± 1	0 - 4
	BXD34_Dnajc3 OE	9	3 ± 2	0 - 8
	B6_GFP	10	0.6 ± 0.3	0.2 - 1.1
Longest 5 axons (mm)	B6 Dnajc3 OE	10	1.1 ± 0.2	0.5 - 1.5
	BXD34_GFP	8	0.5 ± 0.2	0.1 - 0.9
	BXD34_Dnajc3 OE	9	0.8 ± 0.3	0.3 - 1.5
	B6_GFP	10	1.0 ± 0.3	0.5 - 1.5
Longest 1 Axon (mm)	B6 Dnajc3 OE	10	1.6 ± 0.4	0.7 - 2.3
	BXD34_GFP	8	0.8 ± 0.2	0.5 - 1.1
	BXD34_Dnajc3 OE	9	1.4 ± 0.4	0.7 - 2.2

The measurements include the number of axons at 0.5 mm from the crush (Counts at 0.5) and 1mm from the crush site (Counts at 1.0), along with the distance traveled for the 5 longest axons (Longest 5 axons) and the single longest axon (Longest 1 Axon). The number of cases for each strain is shown (n). Measurements are shown as mean ± SD.

The axonal regeneration observed following the knockdown of *Dnajc3* in BXD90, a high regenerative strain, is significantly greater than that observed in BXD18, a low regenerative strain (**Figure 7**). This is the case for the number of regenerating axons as well as the distance the axons travel down the optic nerve. Although the regenerative capacity is significantly diminished in *Dnajc3* knockdown in BXD90 mice relative to the GFP controls (*P* < 0.05), it still does not reach the level of the low regenerative strain, BXD18 (**Figure 7**). These findings indicate that *Dnajc3* modulates optic nerve regeneration in the BXD strain set; however, *Dnajc3* is not solely responsible for the variation in regeneration observed in the BXD strain set, for the increased regeneration is not completely blocked by knocking down *Dnajc3*.

### Overexpression of *Dnajc3*

**Figure 8:**
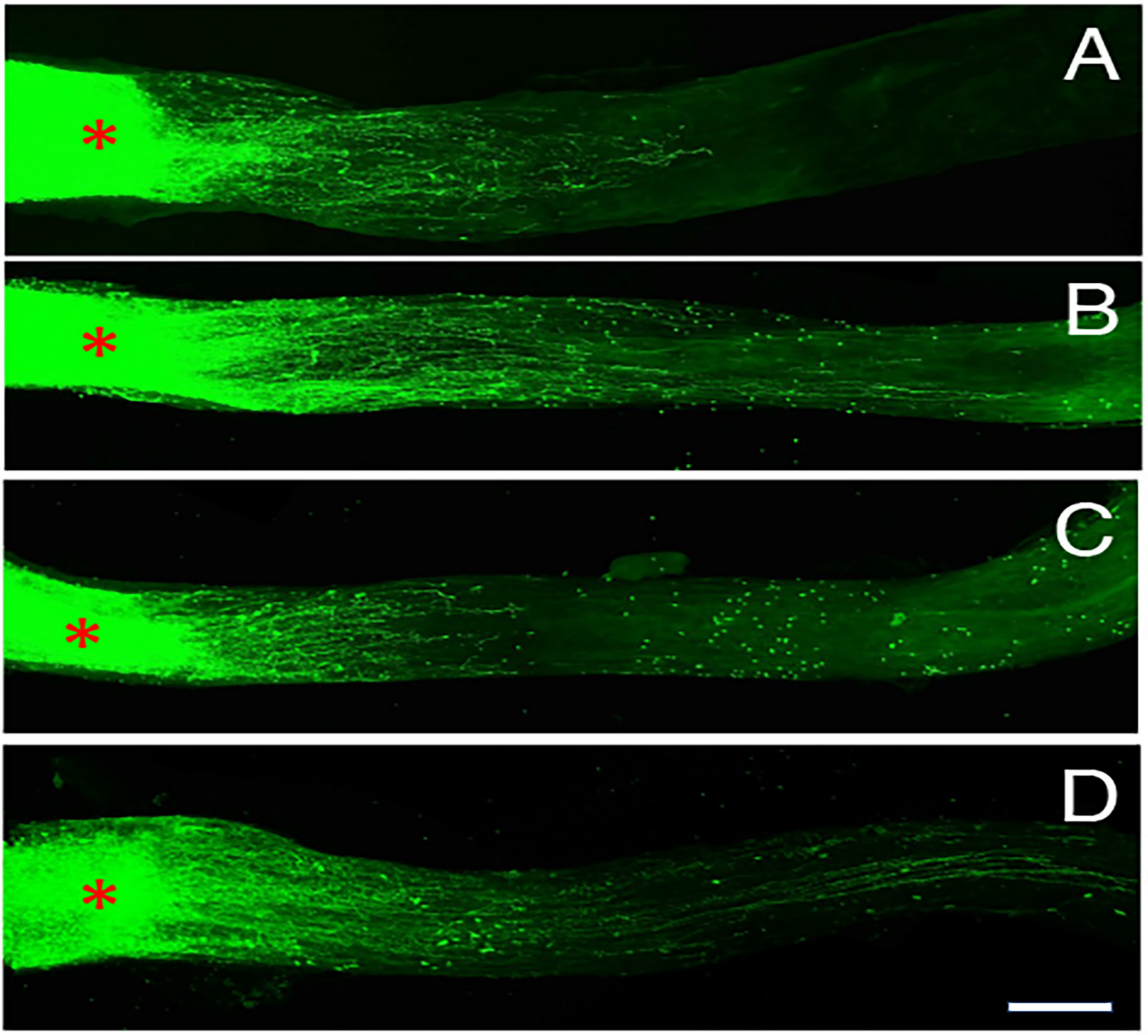
Axon regeneration after *Dnajc3* overexpression. To test for the ability of *Dnajc3* to facilitate axonal regeneration, we over expressed *Dnajc3* in RGCs of two different strains of mice. The regeneration observed in the C57BL/6J strain following transduction with AAV-GFP is shown in A. Over expressing *Dnajc3* in this strain increased the amount of axon regeneration (B). The low regenerating strain BXD34 was also used. The regenerative response observed in BXD34 following transduction with AAV-GFP is illustrated in C. Following over expression of *Dnajc3* an increase in axon regeneration was seen (D). The location of the crush site is designated by a red asterisk. Compared to the retinas transduced with AAV-GFP, there was an increased regeneration in animals in which *Dnajc3* was overexpressed using AAV-Dnajc3. Scale bar: 200 µm. AAV: Adeno-associated virus; Dnajc3: *Dnaj* heat shock protein family member 40; GFP: green fluorescent protein.

**Figure 9:**
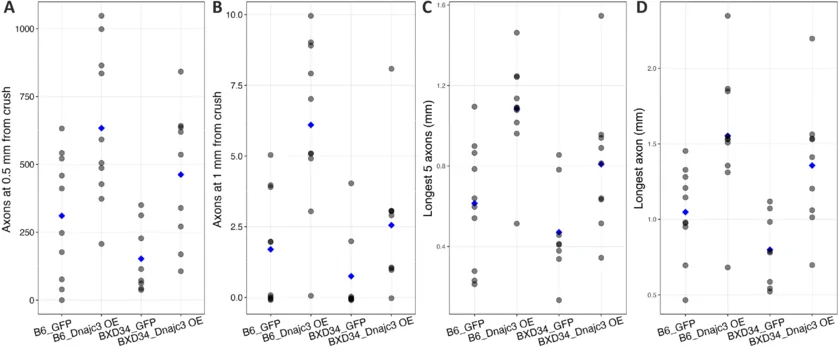
Overexpression of Dnajc3 in C57BL/6J mice and in BXD34 mice. BXD34 was one of the strains with the least optic nerve regeneration induced by our regeneration protocol. *Dnajc3* was over expressed using AAV-*Dnajc3* to express it in RGCs prior to testing for optic nerve regeneration. Each animal is represented by a dot and the means are presented as blue diamonds. In panel A, the number of axons at 0.5 mm from the crush site is shown. The first column is the data from control C57BL/6J mice receiving AAV-GFP (B6_GFP). The effects of overexpressing *Dnajc3* are shown in the next column (B6_*Dnajc3*_OE). There was a significant (*P* < 0.05 Mann-Whitney *U* test) increase in the number of axons when *Dnajc3* was overexpressed. The next column shows the data from the BXD34 GFP control (BXD34_GFP). The data from overexpressing *Dnajc3* in BXD34 is the last column (BXD34_*Dnajc3*_OE). There was a significant increase in the number of regenerating axons (*P* < 0.05 Mann-Whitney *U* test). Similar results are shown for number of axons at 1 mm from the crush site (B). This is also the case for the distance axons regrew down the optic nerve, for both the five longest axons (C) and the longest single axon (D). These data demonstrate that overexpressing *Dnajc3* in a low regenerating strain of either C57BL/6J or BXD34 increases the regeneration of axons following optic nerve crush. AAV: Adeno-associated virus; GFP: green fluorescent protein.

To provide additional evidence that *Dnajc3* modulates axonal regeneration, we used AAV to overexpress *Dnajc3* in the RGCs of one of the parental strains (C57BL/6J) and one of the lowest regenerating strains (BXD34). The full cDNA sequence of *Dnajc3* was placed into a AAV vector and packaged into AAV2 capsid. The AAV-*Dnajc3* was injected into the vitreous of 10 C57BL/6J mice and 9 BXD34 mice, while the controls were injected with AAV-GFP in 10 C57BL/6J mice and in 8 BXD34 mice one week prior to the optic nerve regeneration protocol (similar schedule as shown in **Figure 5A**). The effectiveness of the transduction was examined by staining retinas that received the AAV-*Dnajc3* vector with antibodies directed against HSP40 and comparing them to retinas of the contralateral non-injected eye (**Additional Figure 3**). In the retinas receiving the AAV-*Dnajc3* vector, many cells demonstrated intense staining for HSP40 as compared to the control retinas. Thus, the overexpression of *Dnajc3* successfully produced RGCs expressing high levels of HSP40. The animals receiving intravitreal injections of AAV-*Dnajc3* demonstrated an increase in axonal regeneration. In the C57BL/6J strain (**Figure 8A** and **B**), there were more axons growing a longer distance down the optic nerve. This was also the case for the BXD34 strain (**Figure 8C** and **D**). When the data were quantified (**Figure 9**), both C57BL/6J strain and BXD34 strain, demonstrated a significant increase (*P* < 0.05) in the number of regenerating axons (**Figure 9A** and **B**) and the distance the regenerating axons traveled down the nerve (**Figure 9C** and **D**). Detailed data is shown in **Additional Table 2**. Thus, overexpressing *Dnajc3* in low regenerating strains increased the amount of axon regeneration down the optic nerve. Taken together, the decrease in axonal regeneration following knocking down *Dnajc3* and the increase in regeneration following overexpressing *Dnajc3*, demonstrate that *Dnajc3* is one of the genomic elements modulating axonal regeneration in the BXD strain set.

## Discussion

In the present study, we have used a forward-genetics approach to identify a genomic locus that modulates the regenerative response induced by knocking down *Pten* and creating a mild inflammatory response with Zymosan and CPT-cAMP. This approach identified one locus on Chromosome 14 (115–119 Mb) that modulates optic nerve regeneration. Within this locus, there were 16 annotated genes, two of which were good candidate genes (*Uggt2* and *Dnajc3*). Subsequent testing eliminated *Uggt2* as a modulator of axonal regeneration and demonstrated that changes in the expression levels of *Dnajc3* expression can alter the regenerative response. Knocking down *Dnajc3* in a high regenerating strain of mice decreases axon regeneration; while overexpressing *Dnajc3* in a low regenerating strain increases the ability of axons to regenerate in the optic nerve. In the BXD strain set, *Dnajc3* modulates the regenerative response produced by knocking down Pten and inducing a mild inflammatory response with Zymosan and CPT-cAMP.

*Dnajc3* is expressed in RGCs at relatively high levels and is localized to the endoplasmic reticulum (ER) (Boriushkin et al., 2015). *Dnajc3* encodes p58IPK, also known as HSP40 (Yan et al., 2002; van Huizen et al., 2003). Like many HSPs, HSP40 is an ER chaperone playing a role in the unfolded protein response (UPR) when neurons are stressed (Yan et al., 2002; van Huizen et al., 2003). The UPR plays an essential role in maintaining cellular homeostasis in CNS neurons by activating in stress conditions when there is an accumulation of misfolded or unfolded proteins in the ER. The UPR is a conserved molecular pathway managing the trafficking of proteins through the endoplasmic reticulum and it is associated with chronic inflammatory diseases (Grootjans et al., 2016). Within the eye ER stress is also known to play a prominent role in glaucoma affecting the trabecular meshwork resulting in an increase in intraocular pressure (Peters et al., 2015; Sato et al., 2021; Ying et al., 2021). In many retinal injury models, ER stress is induced and there is an elevation of HSPs as part of the UPR. This includes HSP70 (Dong et al., 2016; Zhang et al., 2023) as well as HSP 40. Unlike many of the chaperone proteins, HSP40 can also function as an inhibitor of eIF2a, a protein kinase localized to the inner surface of the endoplasmic reticulum (Yan et al., 2002). Crucially, in the context of retinal health, the role of HSP40 (p58IPK) in protein homeostasis and anti-inflammatory response has been identified as protective against RGC degeneration (Boriushkin et al., 2015; McLaughlin et al., 2018, 2023), a primary cause of visual impairment in conditions like glaucoma. The protective effect of HSP40 was revealed by Zhang’s group from both *in vitro* (Boriushkin et al., 2015; McLaughlin et al., 2018) and *in vivo* (McLaughlin et al., 2018, 2023) experiments. They reported that deficiency of HSP40 makes the RGCs more susceptible to cell death by the ER stress inducer; while overexpression of p58IPK by AAV increases RGC survival under ER stress (McLaughlin et al., 2018). HSP40 protects RGCs from retinal ischemia/reperfusion and from elevated intraocular pressure caused by microbead injections into the anterior chamber (McLaughlin et al., 2018). Knocking down HSP40 *in vivo* and *in vitro* causes a decrease in RGC survival while overexpressing the protein promotes neuronal survival in culture (McLaughlin et al., 2018). These data strongly support the role of HSP40 in protecting RGCs from stress-induced cell death.

Using the BXD mouse strains, we show that *Dnajc3* is a genomic element modulating axonal regeneration. This includes the number of regenerating axons at 0.5 mm and 1 mm from the crush site, as well as the distance these axons travel down the optic nerve, whether it is the 5 longest axons or the single longest axon. Our results suggest that one potential mechanism responsible for the partial increase in the number of regenerating axons is the neuroprotection of RGC cell body from death. Previous studies (Boriushkin et al., 2015; McLaughlin et al., 2023) demonstrate that overexpressing *Dnajc3* (HSP40) is neuroprotective for RGCs in an elevated intraocular pressure model of glaucoma and in an ischemia/reperfusion model in the mouse. If *Dnajc3* can act as a neuroprotective genomic element, then the increase in the number of regenerating axons could be directly due to the fact that more RGCs are surviving the insult to the optic nerve. Thus, the number of regenerating axons may be directly related to the neuroprotective effects of *Dnajc3* in RGCs; the more RGCs that survive following optic nerve crush, the more axons that are available to regenerate down the optic nerve.

Interestingly, we have found that the genomic loci modulating the distance axons regenerate down the optic nerve are similar to those affecting the number of regenerating axons. Clearly, RGC survival is one of the fundamental aspects necessary for axon regeneration, for if the cell body dies, the cell will not be able to regrow down the optic nerve. That being said, there is increasing evidence that RGC survival and axon regeneration can be two completely independent processes. An example of this is the effect of *Sox11* on retinal injury. The overexpression of *Sox11* in mouse RGCs promotes regeneration (Norsworthy et al., 2017). Interestingly, it does not promote regeneration in some of the RGC subtypes. αRGCs, which are normally associated with induced regeneration down the optic nerve, are killed by *Sox11* overexpression (Norsworthy et al., 2017). Thus, the increased axonal regeneration induced by *Sox11* overexpression must be due to a response from a different RGC subtype, not the αRGCs. The downregulation of *Sox11* increased RGC survival following injury of optic nerve axons (Welsbie et al., 2017; Li et al., 2018). Even though there was an increase in RGC survival, there was not an improved regeneration of axons down the optic nerve in an induced optic regeneration model (Welsbie et al., 2017; Li et al., 2018). Thus, the neuroprotection of RGCs does not result in an obligatory increase in axonal regeneration. That being said, there may be elements that facilitate RGC survival and facilitate axonal regeneration.

Independent of RGC survival, HSP40 (p58IPK) might also influence axon regeneration in the following aspects. In the past, it was believed that while neurons in the peripheral nervous system (PNS) could regenerate after injury, mature neurons in the CNS could not. However, numerous subsequent studies have achieved regeneration of CNS neurons through experimental methods. In the injured PNS, regeneration involves the local synthesis of damage signals and translation of growth-promoting mRNA, such as Gap-43 and β-actin (Donnelly et al., 2011). Under certain conditions, similar mRNA was also detected in injured CNS axons (Kalinski et al., 2015). Local protein synthesis events detected in embryonic axons and adult PNS axons play a pivotal role in neuronal regeneration (Jung et al., 2012). For CNS neurons, axon regeneration requires the fulfillment of several conditions. One critical aspect is the synthesis and transportation of proteins to supply the nutrients and raw materials necessary for axon regeneration. First, protein synthesis is vital for the formation of growth cones. Research has shown that inhibiting protein synthesis impairs growth cones, which are essential structures for axon regeneration (Verma et al., 2005). Second, processes involved in axon regeneration, such as retrograde signaling, growth cone formation, and axon elongation, all depend on local translation (Jung et al., 2012). Adult local transcriptomes detected in RGCs, as well as translation mechanisms in several adult brain regions, have confirmed local translation in mature CNS axons (Shigeoka et al., 2016; Hafner et al., 2019; Ostroff et al., 2019). Moreover, during CNS development, ribosome localization in axons might gradually decrease, indicating that ribosome positioning and mRNA translation specificity change with maturity (Shigeoka et al., 2016; Costa et al., 2019). After injury, the local translation dynamics of adult CNS axons might also decline, such as reduced axonal transcripts or a lack of functional ribosomes (Shigeoka et al., 2019). Furthermore, local translation might be hindered due to incorrect mRNA transport or the retention of mRNA in stress granules (Donnelly et al., 2011; Sahoo et al., 2018). Therefore, it can be inferred that the limited regenerative capacity of CNS axons might be related to reduced local translation. Supporting this, earlier research by Park and colleagues (Park et al., 2008) demonstrated that enhancing protein synthesis by augmenting mTOR signaling activity through silencing the *Pten* gene greatly promotes optic nerve axon regeneration, possibly by influencing local axonal translation (Park et al., 2008). In this experiment, the establishment of the axon regeneration model was based on silencing the *Pten* gene. As a chaperone protein in ER stress, HSP40 may alleviate ER stress, thereby enhancing the protein synthesis capability of the ER as well as the protein translation and transportation abilities at the site of axon injury. This series of effects means that neurons influenced by HSP40 not only have enhanced survival capabilities but also exhibit significantly increased axonal regenerative abilities.

When we examine the effects of altering the expression of *Dnajc3*, it is clear that it is not the sole element affecting the difference in axonal regeneration across the BXD strain examined in the current study. Knocking down *Dnajc3* in a strain with extensive axonal regeneration does not reduce the axonal regrowth to the level of the lower expressing strains. Furthermore, overexpressing *Dnajc3* in a strain with limited axonal regrowth does not enhance the regeneration to the same level as a strain with robust optic nerve regeneration. The regeneration seen in the parental strains demonstrates genetic transgression, where the parental strains lie near the middle of the phenotype distribution. This indicates that at least two genomic elements are modulating the phenotype and axonal regeneration. Taken together, these data that there is at least a second genomic element interacting with *Dnajc3* or directly modulating optic nerve regeneration that differs between the two strains of mice.

## Limitations

This study has limitations that should be noted. We used the BXD strain set to identify *Dnajc3* (encoding HSP40) as a gene modulating axonal regeneration down the optic nerve. In the two parental strains (C57BL/6J and DBA/2J) many of the genes and genomic elements are very similar or identical. Since our analysis can only detect differences in gene expression, the contribution of these similar genomic elements to axonal regrowth cannot be detected. We can only monitor approximately 15% of genome that differ between the C57BL/6J mouse and the DBA/2J mouse. Thus, many contributions of other genomic loci remain unknown.

## Conclusions

Optic nerve regeneration is a highly complex process with many factors interacting and influencing each other. Using a forward-genetics approach, we identified *Dnajc3* as a genomic element promoting an increase in the number of regenerating axons as well as the distance these axons travel. The protein it encodes, HSP40, is one element modulating axonal regeneration in the mouse. HSP40 is an important factor within the RGCs. Overexpressing or knocking down HSP40 affects the regenerative capacity of injured RGCs. One of the underlying mechanisms in this increased regeneration is the role of HSP40 in protecting RGCs from death. In addition, it improves the ability of neurons to overcome the unfolded protein response that complicates axon regrowth. In the future, it may be possible to modulate the actions of HSP40 pharmacologically to improve the regenerative capacity in the optic nerve and this may prove to be critical to functional regeneration in humans as the distance the axons have to travel to reach their targets in the human brain is considerably longer than that in most rodent models.

## Additional files:

***Additional Figure 1:*** *Knockdown of Pten affecting the mTOR pathway in retinal ganglion cells.*

Additional Figure 1 Knockdown of Pten affecting the mTOR pathway in retinal ganglion cells.
To determine if knocking down Pten in our experiments caused an increase in phosphorylated S6, retinas from an AAV-sh*Pten*-GFP transduced retina (A-D) and from an AAV-GFP transduced retina (E and F) were stained red with antibodies directed against S6 (A, B, E, F) or pS6 (C, D, G, H). GFP-positive cells were immunostained for GFP (B, D, F, H) and all nuclei were stained with TOPRO (Blue). Notice that virtually all cells in ganglion cell layer are positive for S6 (A, B, E, F). In the retina transduced with AAV-sh*Pten*-GFP (C, D) many of the neurons are positive for pS6 and cells expressing GFP can also be seen expressing pS6 (arrows in C and D). When the retinas were transduced with AAV-GFP there is little staining for pS6 and the transduced cells in H are not showing colocalization with pS6-positive cells. These data reveal that the AAV-sh*Pten*-GFP transduced cells are indeed affecting the mTOR pathway by knocking down *Pten*. Scale bar: 50 μm. AAV: Adeno-associated virus; GFP: green fluorescent protein.

***Additional Figure 2:*** *Genome-wide interval maps for axon regeneration.*

Additional Figure 2 Genome-wide interval maps for axon regeneration.
Genome-wide interval maps for the number of axons at 0.5 mm from the crush site (A) and the number of axons 1mm from the crush site (B) are shown. In addition, the distance traveled by the longest five regenerating axons (C) and the longest single axon (D) are illustrated. The interval map plots the likelihood ratio statistic (LRS) across the genome from chromosome 1 to chromosome X. The light gray line is the suggestive level and the light red line is the genome-wide significance (*P*=0.05). When the optic nerve regeneration measures were mapped to the mouse genome, a significant association between regeneration and a locus on Chromosome 14 was observed.

***Additional Figure 3:*** *Overexpression of Dnajc2.*

Additional Figure 3 Overexpression of *Dnajc2*.
Photomicrographs of retinas injected with AAV-*Dnajc3* and stained for HSP40 (B) and the non-injected retina served as a control (A). In the control retina the presence of HSP40 (green) can be seen within the RBPMS-stained RGCs (red). Nuclei stained with TOPRO are shown in blue. In B the intense green staining of RGCs transduced with AAV-*Dnajc3* is illustrated. When we scanned the retina over expressing Dnajc3 the photomultiplier was completely saturated. For the image in B the lasers and sensitivity of the detectors had to be turned down. We used these same settings to take the image illustrated in A. Scale bar: 50 μm. AAV: Adeno-associated virus; HSP40: heat shock protein 40; RGC: retinal ganglion cell.

***Additional Figure 4:*** *Vector map for AAV-shPten-GFP vector.*

Additional Figure 4 Vector map for AAV-sh*Pten*-GFP vector.
The Vector map for the AAV-sh*Pten*-GFP is shown along with the sequence of the entire vector. AAV: Adeno-associated virus; GFP: green fluorescent protein.

***Additional Figure 5:*** *Vector map for AAV-shUggt2-GFP vector.*

Additional Figure 5 Vector map for AAV-sh*Uggt2*-GFP vector.
The Vector map for the AAV-sh*Uggt2*-GFP is shown along with the sequence of the entire vector. AAV: Adeno-associated virus; GFP: green fluorescent protein.

***Additional Figure 6:*** *Vector map for AAV-Dnajc3 vector.*

Additional Figure 6 Vector map for AAV-*Dnajc3* vector.
The Vector map for the AAV-*Dnajc* is shown along with the sequence of the entire vector. AAV: Adeno-associated virus.

***Additional Figure 7:*** *Vector map for AAV-shDnajc32-GFP vector.*

Additional Figure 7 Vector map for AAV-sh*Dnajc32*-GFP vector.
The Vector map for the AAV-sh*Dnajc3*-GFP is shown along with the sequence of the entire vector. AAV: Adeno-associated virus; GFP: green fluorescent protein.

***Additional Table 1:*** *Measurements used to quantify axonal regeneration in BXD mouse strains (Strains).*

***Additional Table 2:*** *Measurements used to quantify axonal regeneration following knockdown of Dnajc3 and Uggt2, and overexpression of Dnajc3.*

## Data Availability

*The data quantifying axonal regeneration in the BXD mouse strain set is available on GeneNetwork (genenetwork.org): number of axons at 0.5mm (BXD_27559), number of axons at 1 mm (BXD_27560), the distance traveled by the 5 longest axons (BXD_27561) and the length of the single longest axon (BXD_27562). All vectors used in this study are available upon request (for Vector Maps and Sequences see Additional Figures 4–7).*
